# Adverse events associated with administration of vasopressor medications through a peripheral intravenous catheter: a systematic review and meta-analysis

**DOI:** 10.1186/s13054-021-03553-1

**Published:** 2021-04-16

**Authors:** Victoria S. Owen, Brianna K. Rosgen, Stephana J. Cherak, Andre Ferland, Henry T. Stelfox, Kirsten M. Fiest, Daniel J. Niven

**Affiliations:** 1 Department of Community Health Sciences, Cumming School of Medicine, University of Calgary, Calgary, Canada; 2 Department of Critical Care Medicine, Ground Floor, McCaig Tower, Cumming School of Medicine, University of Calgary, 3134 Hospital Drive NW, Calgary, AB T2N 5A1 Canada; 3 O’Brien Institute for Public Health, Cumming School of Medicine, University of Calgary, Calgary, Canada

**Keywords:** Peripheral intravenous, Vasopressor, Vasoconstrictor, Ionotrope, Adverse event, Safety

## Abstract

**Background:**

It is unclear whether vasopressors can be safely administered through a peripheral intravenous (PIV). Systematic review and meta-analysis methodology was used to examine the incidence of local anatomic adverse events associated with PIV vasopressor administration in patients of any age cared for in any acute care environment.

**Methods:**

MEDLINE, EMBASE, CINAHL, the Cochrane Central Register of controlled trials, and the Database of Abstracts of Reviews of Effects were searched without restriction from inception to October 2019. References of included studies and related reviews, as well as relevant conference proceedings were also searched. Studies were included if they were: (1) cohort, quasi-experimental, or randomized controlled trial study design; (2) conducted in humans of any age or clinical setting; and (3) reported on local anatomic adverse events associated with PIV vasopressor administration. Risk of bias was assessed using the Revised Cochrane risk-of-bias tool for randomized trials or the Joanna Briggs Institute checklist for prevalence studies where appropriate. Incidence estimates were pooled using random effects meta-analysis. Subgroup analyses were used to explore sources of heterogeneity.

**Results:**

Twenty-three studies were included in the systematic review, of which 16 and 7 described adults and children, respectively. Meta-analysis from 11 adult studies including 16,055 patients demonstrated a pooled incidence proportion of adverse events associated with PIV vasopressor administration as 1.8% (95% CI 0.1–4.8%, *I*^2^ = 93.7%). In children, meta-analysis from four studies and 388 patients demonstrated a pooled incidence proportion of adverse events as 3.3% (95% CI 0.0–10.1%, *I*^2^ = 82.4%). Subgroup analyses did not detect any statistically significant effects associated with stratification based on differences in clinical location, risk of bias or design between studies, PIV location and size, or vasopressor type or duration. Most studies had high or some concern for risk of bias.

**Conclusion:**

The incidence of adverse events associated with PIV vasopressor administration is low. Additional research is required to examine the effects of PIV location and size, vasopressor type and dose, and patient characteristics on the safety of PIV vasopressor administration.

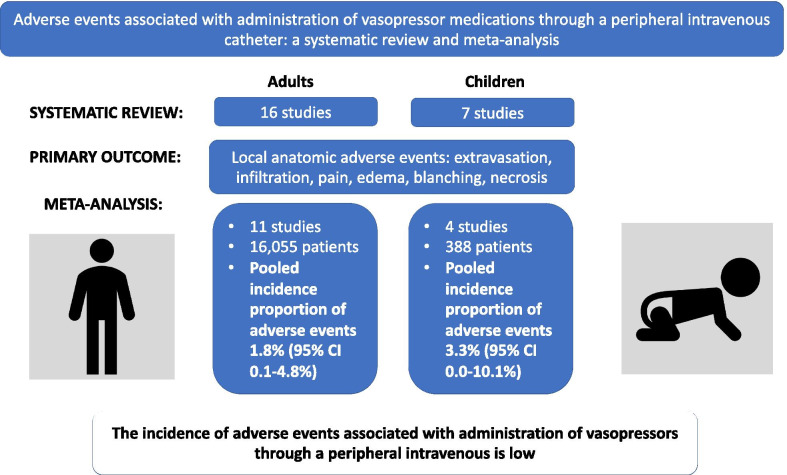

**Supplementary Information:**

The online version contains supplementary material available at 10.1186/s13054-021-03553-1.

## Background

Intravenous vasopressors are commonly used to augment systemic blood pressure [1]. Current practice, especially within intensive care units (ICUs), is to administer these medications through a central venous catheter (CVC) [2, 3]. The reason for preferential use of CVCs rather than the more ubiquitous peripheral intravenous (PIV) catheters is due to the long-standing concern regarding vasopressor extravasation outside the PIV and potential for tissue damage (e.g. infiltration, ischemia and necrosis) [1, 4]. However, insertion and maintenance of a CVC is not without risk. A recent study examining CVCs in adults found that up to 2.1% of patients experienced significant mechanical complications such as pneumothorax requiring intervention, 0.5–1.4% experienced symptomatic deep-vein thrombosis directly attributable to the CVC, and another 0.5–1.4% experienced bloodstream infection [5]. Current estimates are similar, if not higher among children [6, 7]. In addition, CVC insertion can be time consuming and is associated with patient discomfort. For many patients, CVC insertion is a necessary intervention. However, for those that do not necessarily require a CVC, the adverse event profile associated with PIV vasopressor administration is less clear.

A previous systematic review of predominantly case reports and case series found that among 263 patients who received vasopressors through a PIV there were 318 adverse events, of which 86 were extravasation with no injury and 179 were skin necrosis [4]. Local tissue injury tended to occur in patients who had vasopressors infused for longer durations of time (≥ 24 h) through a PIV distal to the popliteal or antecubital fossa [4]. However, case series and case reports are at high risk for reporting bias [8], therefore the rate of adverse events associated with PIV vasopressor administration could not be estimated. In addition, larger cohort studies examining adverse events associated with PIV vasopressor administration in adults [2, 3, 9–11] have since been published as well as multiple recent studies in the child population [12–14]. We used systematic review and meta-analysis methodology to examine the incidence of adverse events associated with vasopressor administration through PIVs in patients of any age cared for in any acute care setting.

## Methods

### Overview and definitions

This study was conducted and reported according to Preferred Reporting Items for Systematic Reviews and Meta-analyses (PRISMA) guidelines [15] and registered with PROSPERO (ID CRD42020155218). A vasopressor was defined as any drug administered intravenously that causes constriction of blood vessels [1]. PIVs were defined as catheters inserted into and terminating in peripheral veins (e.g., arms or legs). This included the midline catheter. CVCs were defined as catheters inserted into large proximal veins wherein the catheter tip terminated within the central circulation [5]. This included internal jugular, subclavian, and femoral sites, as well as the peripherally inserted CVC, i.e. PICC. Adverse events were as defined by each individual study but had to be local anatomic events directly attributable to the administration of a vasopressor infusion through a PIV (e.g., extravasation, infiltration, necrosis) and not related to its potential systemic effects (e.g. tachyarrhythmia). For the purpose of this review, a cohort study was defined as a study that followed participants from exposure to outcome, had greater than 10 participants, and investigated patients who experienced and did not experience an adverse event.

### Search strategy

The search strategy was developed in consultation with a medical librarian (see acknowledgements) and peer reviewed using the Peer Reviewed of Electronic Search Strategies checklist (Additional file 1: Table S1) [16]. The following electronic databases were searched from inception to October 16, 2019: MEDLINE (OVID), EMBASE (OVID), CINAHL (EBSCO), Cochrane Central Register of Controlled Trials (OVID), and the Database of Abstracts of Reviews of Effects (OVID). No restrictions were placed on language, date, or country of publication. Additional searches were conducted within reference lists of included studies, relevant reviews, and proceedings from relevant conferences (Additional file 1: Table S2).

### Citation screening and eligibility criteria

Screening of titles and abstracts as well as full text articles was conducted independently and in duplicate using DistillerSR (Evidence Partners, Ottawa, Canada). At the title and abstract level, if either reviewer (VSO, BKR) deemed a citation potentially relevant it progressed to more detailed review at full text screening (VSO, BKR, SJC). Disagreements were resolved by discussion or involvement of an additional reviewer (DJN). For articles not written in English, Google Translate was used [17] and if the article was not readable by Google Translate a person fluent in the language translated the article.

Studies were included in the systematic review if they met the following eligibility criteria: (1) cohort, quasi-experimental, or randomized controlled trial design; (2) examined continuous vasopressor infusion administration through PIVs in humans (any age or clinical setting); and (3) investigated local anatomic adverse events associated with PIV vasopressor administration. Studies were subsequently included in the meta-analysis if they reported (1) data required to calculate the incidence of adverse events per patient (i.e., both the number at risk, and the number that experienced adverse events) and (2) the PIVs were inserted and managed in a manner consistent with contemporary clinical practices.

### Data extraction and risk of bias estimation

Data was extracted independently and in duplicate using a standardized form developed in Microsoft Excel. For studies where important data was missing, corresponding authors were contacted. Extracted data included study characteristics, participant characteristics and adverse events. The primary outcome was the incidence proportion per patient of local anatomic adverse events associated with PIV vasopressor administration. This incidence proportion was calculated by dividing the number of patients who experienced a local anatomical adverse event by the number of patients prescribed PIV vasopressors.

Risk of bias was assessed independently and in duplicate by two reviewers (VSO, BKR) using the Revised Cochrane risk-of-bias tool for randomized trials (RoB 2) [18] or the Joanna Briggs Institute (JBI) checklist for prevalence studies [19] where appropriate. Disagreements were resolved by consensus, or consultation with an additional reviewer (DJN). As done with previous literature, for studies evaluated using the JBI checklist, responses were tallied to generate an overall risk of bias for each study [20, 21]. Studies at or below the median score were deemed to have some concern or high risk of bias, whereas those above the median were low risk. Results of the two tools were dichotomized into low versus high/some concern for risk of bias.

### Statistical analysis

The pooled incidence proportion of adverse events associated with PIV vasopressor administration was estimated using a random effects model and the ‘metaprop’ package [22] in Stata version 16.1 (StataCorp, College Station, TX). Data from studies in adults were pooled separately to those in children. Statistical heterogeneity was assessed using the *I*^2^ statistic and Cochran’s *Q* test. Since proportion estimates were at or close to the border of permissible values (no or very few adverse events reported), the Freeman–Tukey double arcsine transformation was used for the pooled estimate [22]. The exact binomial method was used to calculate the study specific confidence intervals which guarantees admissible values [22]. Possible sources of heterogeneity were examined using sub-group analyses and metaprop’s test of heterogeneity between groups [22]. Publication bias was assessed through inspection of a funnel plot and Begg’s test. Statistical significance was denoted by *p* < 0.05.

## Results

### Study selection

**Fig. 1:**
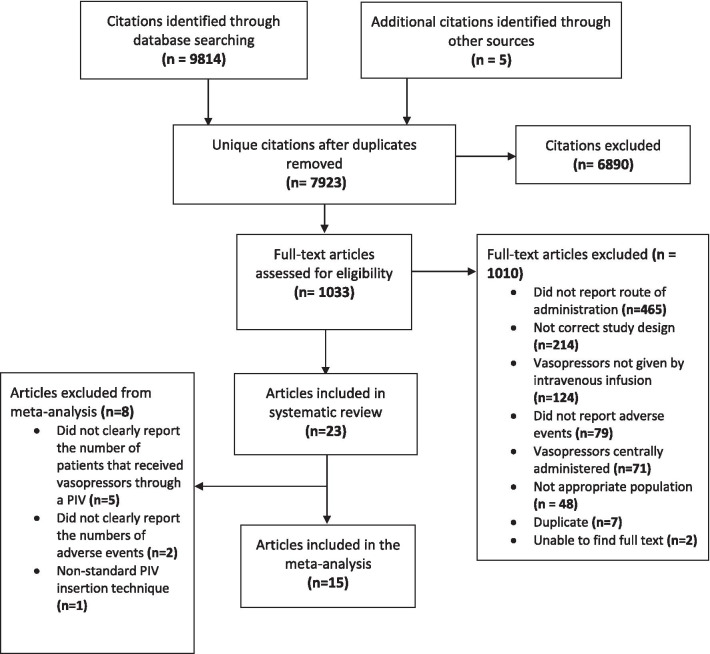
PRISMA flow diagram

The search strategy identified 9814 citations, from which 7923 unique citations were screened for inclusion (Fig. 1). Detailed full text review of 1,033 studies (kappa = 0.92) resulted in 23 studies (all written in the English language) included in the final systematic review [2, 3, 9–14, 23–37], and 15 of those in the meta-analysis [2, 3, 9–13, 30–37]. Studies excluded after full text review commonly did not report the route of vasopressor administration (*n* = 465, 46%), or did not employ the desired study design (*n* = 214, 21%). Of the eight articles excluded from the meta-analysis, the most common deficiency related to not clearly reporting the number of patients that received vasopressors through a PIV (*n* = 5) [14, 25–28]. Other reasons for exclusion from the meta-analysis were failure to clearly report the numbers of adverse events (*n* = 2) [23, 29], and outdated PIV insertion technique (*n* = 1) [24]. Two recent studies among children were not included in the meta-analysis due to inability to distinguish between PIV and interosseous (IO) administration of vasopressors. Adverse events were very low or absent in these studies [14, 33].

### Study characteristics

Of the 23 studies in the systematic review, 16 were in adults, and seven were in children (Table 1). Studies were primarily cohort studies (*n* = 18, 78%), but also included randomized control trials (RCTs) (*n* = 4, 17%). Of the cohort studies, most were retrospective (*n* = 10, 43%). Among the 16 adult studies, five (31%) described patients in emergency departments (ED), six (37%) described patients in ICUs, and the other five (31%) included patients from a mixture of clinical settings. Among children, clinical settings included the pediatric intensive care unit (PICU, *n* = 2, 29%), neonatal intensive care unit (NICU, *n* = 2, 29%), and mixture of ED and PICU (*n* = 2, 29%).

**Table 1:** Characteristics of included studies

Author (year)	Study location	Location of care	Study design	Patients (*n*)	Received PIV vasopressors (*n*)	Adverse events associated with PIV vasopressors^a^ (*n*)	Included in meta-analysis
*Studies in adults*							
Andrews [35]	Zambia	ED	RCT^b^	209	17^c^	0	Yes
Johnson, W. [30]	USA^d^	Surgical ICU	RCT^e^	25	11	1	Yes
Medlej [10]	Lebanon^d^	ED/ICU	Prospective cohort	55	55	3	Yes
Lewis [3]	USA	Medical ICU/ step down unit	Retrospective cohort	202	202^f^	8	Yes
Pancaro [11]	Netherlands	OR	Retrospective cohort	14,385	14,385	5	Yes
Datar [9]	USA	Neuro ICU	Retrospective cohort	277	277	9	Yes
Hallengren [36]	Sweden	Intermediate care unit	Retrospective cohort	91	79	0	Yes
Delgado [34] ^g^	USA	Neuro ICU	Retrospective cohort	20	20	1	Yes
Cardenas-Garcia [2]	USA	Medical ICU	Combined prospective /retrospective cohort	734	734^h^	19	Yes
Putland [31]	Australia	ED	Retrospective cohort	220	220	7^i^	Yes
Rojewski-Rojas [37]^j,k^	Spain	ED	Retrospective cohort	55	55	2	Yes
Delaney [29]	Multinational^l^	ED	Post-hoc analysis of RCT^m^	937	389	–^n^	No^o^
White [28]	USA	Hospital^p^	Prospective cohort	188	13^q^	1	No^q^
Dugger [27]^k^	USA	Hospital^p^	Combined prospective /retrospective cohort	25^r^	25^r^	17^r^	No ^q^
Zucker [24]^k^	USA	Hospital^p^	Cohort study^s^	68	68	22^t^	No^u^
Moyer [23]	USA	Hospital^p^	Cohort study^s^	20	20	–^o^	No^o^
*Studies in children*							
Kumar [12]	India	Pediatric ICU/ED	Prospective cohort	204	190	3	Yes
Patregnani [13]	USA	Pediatric ICU	Retrospective cohort	102	102	2	Yes
Turner [32]	USA	Transport to Pediatric ICU	Retrospective cohort	73	73	11	Yes
Lampin [33]	France	Pediatric ICU	Retrospective cohort	144	23^v^	0^v^	Yes^v^
Ventura [14]	Brazil	ED/Pediatric ICU	RCT^w^	120	–^w^	0	No^r^
Stanley [26]	USA	Neonatal ICU	RCT^x^	772^s^	–^q^	–^o^	No^o,q^
Johnson [25]	USA	Neonatal ICU	Prospective cohort	69	– ^q^	1	No ^q^

^a^Local anatomic adverse events as defined by each individual study

^b^RCT examining outcomes between septic patients admitted to the ED

^c^Adverse events were only monitored for within the first 6 h of admission to ED

^d^Implied

^e^RCT examining routes of vasopressin administration (peripheral and superior mesenteric artery)

^f^340 PIVs administering vasopressors in 202 patients

^g^Pilot study with an age range of 14–90 years

^h^783 infusions in 734 patients. 49 patients had more than one PIV infusions

^i^Study had 11 cases of local tissue ischemia happened in 7 patients (5 in same patient)

^j^Study abstract with poster only. Data preferentially taken from poster

^k^Implied adult population

^l^Most of the 51 centers included in original RCT were in Australia or New Zealand

^m^Used data from ARISE trial [45]

^n^Adverse events related to administration of vasopressors through a PIV not recorded in original RCT

^o^Does not clearly report the numbers of adverse events

^p^Unclear specific location of care within the hospital

^q^Does not clearly report the number of patients that received vasopressors through a PIV

^r^Number of infusions, could be more infusions than patients

^s^Unclear if data was collected prospectively or retrospectively

^t^Study had 34 extravasations happened in 22 patients

^u^Reports adverse events with non-standard PIV insertion technique (cut downs to cannulate veins)

^v^Study reports 1 case of PIV dopamine extravasation causing ischemia and skin necrosis, but as it does not report total number of PIV dopamine. Numbers included in meta-analysis are only for epinephrine

^w^Patients were randomly assigned to either received dopamine or epinephrine through a PIV or IO in fluid-refractory septic shock. No stratification was done between PIV and IO administration

^x^Patients randomized between vialon and Teflon catheters

### Characteristics of patients that received PIV vasopressors

The characteristics of patients who received PIV vasopressors are presented in Additional file 1: Table S3. In the 10 adult studies that reported age and sex, average (mean or median) age ranged from 36 to 81 years and most study participants were male (1089/2051, 53%). In adult studies, norepinephrine was the most commonly administered vasopressor (15,584 cases in 9 studies), followed by phenylephrine (546 cases in 4 studies), epinephrine (261 cases in 4 studies), and dopamine (151 cases in 6 studies). Average PIV vasopressor infusion duration was reported in 10 adult studies, and ranged from 1.3 to 49 h, with half (*n* = 5) of studies reporting an average duration between 12 and 24 h. In the six adult studies that reported the size of the PIV used to administer vasopressors, 88% of PIV vasopressor infusions were through PIVs that were 16–20 gauge (1283/1464), of which most were through a 20-gauge PIV (888/1464, 61%). Seven adult studies commented on the anatomical location of PIV, of which four quantified how many infusions were recorded through each location. From these studies, 64% (446/692) of vasopressors were administered through a PIV in the arm proximal to the wrist and 27% (188/692) were administered in the hand or wrist.

In the seven studies done in children, the average age ranged from 1 day to 9.3 years. Dopamine was the most commonly administered vasopressor and was administered to patients in all of the child studies (*n* = 7), followed by epinephrine (*n* = 4 studies), and norepinephrine (*n* = 3 studies). Most of the studies in children (*n* = 5) described patients receiving multiple vasopressors, with one study explicitly describing patients receiving multiple vasopressors simultaneously through the same PIV [13]. Average duration of infusion was reported in five child studies and ranged from 3 to 36.5 h, with most (*n* = 3 studies) reporting a duration under six hours. Gauge was reported in four studies with 24 gauge as most common PIV size.

### Adverse events associated with PIV vasopressors

Adverse events associated with PIV vasopressor administration are described in Additional file 1: Table S3, with characteristics of those that experienced adverse events in Additional file 1: Table S4. Twenty studies explicitly examined for the occurrence of severe adverse events (e.g., necrosis or limb ischemia). Nineteen studies reported any adverse event including those that were mild, of which four studies reported severe adverse events that included thrombophlebitis (one case [10]), slough (two cases [24, 25]), and ischemia with skin necrosis (one case [33]). The other 15 studies that observed adverse events described infiltration or extravasation leading to mild local tissue reactions such as edema, erythema, blanching, phlebitis, discomfort or mottling. Cutaneous discoloration (*n* = 2 patients) was the only long term complication [12] reported in the studies observing more mild adverse events.

Of the 19 studies that reported adverse events, seven reported the resolution of adverse events without treatment (other than removal of PIV), four reported minor treatment (e.g. phentolamine injection and nitroglycerin paste application), and the other eight studies did not mention use of any treatment. In one adult study, three patients had PIV access challenges (e.g., problems with PIV infusing or obtaining PIV site) which were resolved by obtaining a new PIV site in one patient and inserting a CVC in the other two patients [36]. One study in children reported four instances of loss of a PIV, but no associated adverse events [13].

**Fig. 2:**
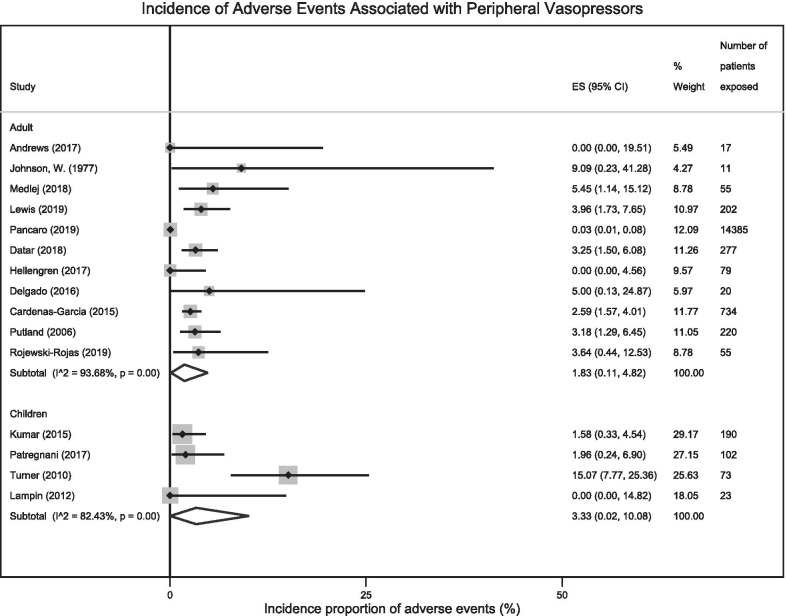
Forest plot examining the incidence proportion of adverse events associated with peripheral intravenous vasopressor administration

Random effects meta-analysis pooling data from 11 adult studies (*n* = 16,055 patients) estimated the incidence proportion of adverse events associated with PIV vasopressor administration as 1.8% (95% CI 0.1–4.8%, *I*^2^ = 93.7%, Cochran’s *Q* *p* < 0.001) (Fig. 2). Pooling data from the four studies in children (*n* = 388 patients) estimated the incidence proportion of adverse events as 3.3% (95% CI 0.0–10.1%, *I*^2^ = 82.4%, Cochran’s Q *p* < 0.001) (Fig. 2). The funnel plot associated with the meta-analysis is in Additional file 1: Fig. S1. Begg’s test did not demonstrate evidence of publication bias (*p *= 0.37).

### Risk of bias assessments

Of the four RCTs assessed by the RoB 2 [18] tool, two had some concerns and two were at high risk of bias (Additional file 1: Table S5). For the 19 observational studies that were assessed using the Joanna Briggs Institute checklist for prevalence studies [19], five studies were at a higher risk of bias than the median, eight studies scored the median and six studies scored lower than the median risk of bias Additional file 1: Table S6).

### Exploration for sources of heterogeneity

**Fig. 3:**
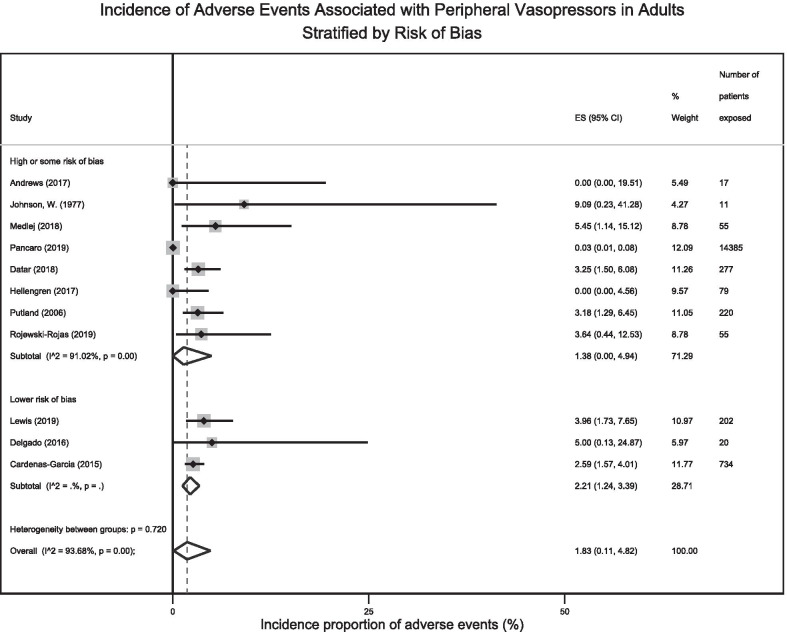
Forest plot of adult incidence proportion of adverse events stratified by risk of bias score. *For Lower Risk of Bias the subtotal *I*^2^ = 0.00%, *p *= 0.40

Subgroup and meta-regression analyses exploring sources of heterogeneity focused on adults owing to a lack of sufficient data to conduct such analyses among children. The subgroups investigated were defined by: location of care, risk of bias, study design, vasopressor infusion duration, PIV gauge, anatomical location of infusion, vasopressor type, and patient sex. Incidence estimates for adverse events associated with PIV vasopressor administration did not differ significantly when examined in subgroups (Table 2, Fig. 3 and Additional file 1: Figs. S2–S8), though heterogeneity varied considerably according to differences in clinical location of care, PIV gauge, vasopressor infusion duration, and risk for methodological bias. Owing to limitations in the data it was not possible to examine subgroups defined by drug concentration or dose.

**Table 2:** Subgroup analyses exploring for sources of heterogeneity between adult studies

Characteristics	Studies (*n*)	Cumulative number of patients	Pooled incidence proportion of adverse events (95% CI)	*I*^2^ (%)	Test of heterogeneity between groups
*Clinical location*					
Shorter stay Units (OR/ED)	5	14,732	1.47% (0.00–6.40%)	91%	*p *= 0.75
Longer stay Units (ICU/Stepdown)	6	1323	1.85% (0.67–3.42%)	37%	
*Risk of bias*					
High or some risk of bias	8	15,099	1.38% (0.00–4.94%)	91%	*p *= 0.72
Lower risk of bias	3	956	2.21% (1.24–3.39%)	0%	
*Study design*					
Randomized	2	28	1.77% (0.00–12.37%)	–	*p *= 0.62
Non-randomized	9	16,027	2.09% (0.24–5.18%)	95%	
*Duration of infusion (mean or median of study)*					
Less than 24 h	8	15,255	1.57% (0.00–5.06%)	93%	*p *= 0.73
Greater or equal to 24 h	3	800	1.50% (0.37–3.11%)	5%	
PIV Gauge^a^					
16-20G	4	1051	2.04% (1.04–3.27%)	6%	*p *= 0.31
22G or smaller	3	105	8.50% (0.00–90.63%)	69%	
Anatomical location^a^					
Hand	2	101	3.05% (0.21–7.92%)	–	*p *= 0.42
Proximal to wrist	3	307	1.19% (0.00–5.13%)	39%	
Vasopressor type^a^					
Norepinephrine	5	15,166	1.40% (0.00–5.13%)	95%	*p *= 0.42
Phenylephrine	4	546	2.03% (0.00–6.59%)	79%	
Epinephrine	2	222	0.00% 0.00–1.57%)	–	
Dopamine	4	125	0.00% (0.00–1.33%)	0%	
Vasopressin	2	15	4.57% (0.00–25.52%)	–	
Sex^a^					
Female	5	302	2.77% (0.44–6.36%)	28%	*p *= 0.43
Male	5	331	1.76% (0.35–3.86%)	0%	

*OR* operating room, *ED* emergency department, *ICU* intensive care unit, *PIV* peripheral intravenous

^a^Analysis involves patients from the same study in multiple different categories. This was only possible for studies that provided the stratification data for both those exposed to PIV vasopressors and those that experienced adverse events with PIV vasopressors

Although we used a random effects meta-analysis to pool data where weighting of included studies is generally distributed evenly, we performed a sensitivity analysis excluding the largest study (*n* = 14,385) of operative patients where event rates appeared to be lower to examine whether its inclusion was driving the main result for the adult studies [11]. The pooled estimate of the incidence proportion of adverse events associated with PIV vasopressor administration among the 10 remaining adult studies (*n* = 1670 patients wherein location of care was primarily ICU and/or ED) was 2.1% (95% CI 1.2–3.1%) with a marked decrease in interstudy heterogeneity (*I*^2^ = 9.21%, Cochran’s Q *p *= 0.36) compared to the main analysis (Additional file 1: Fig. S9).

## Discussion

This systematic review and meta-analysis of adverse events associated with PIV vasopressor administration among more than 16,000 patients from 23 studies found that in both adults and children, the risk of local anatomic adverse events is low and comparable to the rates of potentially more serious complications associated with CVC insertion and maintenance. The pooled incidence of adverse events was 1.8% (95% CI 0.1–4.8%) among adults and 3.3% (95% CI 0.0–10.1%) among children. In addition to the generally low incidence, 19 of the 23 studies reported either no adverse events or only mild local tissue reactions like infiltration or extravasation, with only one study [33] reporting ischemia and skin necrosis. Importantly, a large number of studies were at some to high risk of bias, and there was significant statistical and clinical inter-study heterogeneity. In addition, although most studies reported PIV vasopressor administration that follows contemporary practice (i.e. PIVs 20 gauge or larger in veins proximal to the hands), no study directly compared the incidence of local anatomical adverse events associated with PIV compared to CVC-administered vasopressors. Therefore, the results of this meta-analysis should be interpreted as supporting the hypothesis that for many patients, PIV vasopressor administration may be safe, whilst also highlighting the need for additional high-quality research.

Our results are consistent with prior literature examining the risks associated with PIV vasopressor administration. A prior systematic review that could not estimate incidence due to the inclusion of case series and case reports suggested adverse events appear associated with longer duration of infusion and distal PIV sites [4]. Generalization of these results to modern practice regarding vasopressor and PIV management was limited by the fact that most included articles were published before 1970 [4]. More recently, Tian et al. examined adverse events associated with PIV vasopressor administration reported by seven cohort studies published between 2010 and 2018 that included 1362 adult patients in shock [38]. Most included patients were admitted to ICUs. They reported a pooled extravasation rate of 3.4% (95% CI 2.5–4.6%) with none causing limb ischemia or tissue necrosis. We included 23 studies, screening all and including five of those in the systematic review of Tian et al. but also included an additional 18 studies in patients cared for outside ICUs in the ED and OR, as well as children. Our pooled adverse event rate for adults was similar to that of Tian et al. [38]. Our pooled rate for children confirms that similar to adults, the incidence of adverse events associated with PIV vasopressor administration is low. To our knowledge, this is the first meta-analysis of PIV vasopressor administration in children.

While the incidence of adverse events associated with PIV vasopressor administration is low, it is difficult to make clinical recommendations without direct comparisons to current practice through CVCs. Designing a study to make those comparisons faces several challenges. First is defining the primary outcome of interest, wherein the adverse events associated with vasopressor administration are different for PIVs and CVCs. Medication extravasation, local tissue ischemia or sudden loss of intravenous access resulting in severe hypotension are events that would be more commonly associated with vasopressors administered through a PIV. Similarly, pneumothorax, arterial puncture, and catheter-related bloodstream infection are more commonly associated with CVCs. In an RCT done by Ricard et al., comparing all-purpose initial access with a PIV (not just for vasopressor administration) to a CVC in 263 patients admitted to ICUs in France, major complications were more common in those initially treated with a PIV [39]. However, greater than five attempts to insert a PIV and pneumothorax owing to CVC insertion were counted equally as major adverse events, which arguably have differing clinical implications [40]. The second challenge in designing an RCT to examine PIV vasopressor administration is the low rate of adverse events associated with administration of vasopressors through either a PIV or CVC that would necessitate a large sample size. Third, is the interaction of the current research question comparing use of PIVs and CVCs to also include newer, more frequently utilized forms of venous access such as PICC and midline catheters, with a midline functioning more like a PIV and the PICC more like a CVC. At first glance these two forms of venous access seem more robust than a PIV and lower risk and discomfort to patients than the traditional CVC. However, recent research suggests that critically ill patients may be higher risk for PICC-related complications such as catheter-related and deep vein thrombus [41]. None of the studies included in this review examined patients managed with midline or PICC catheters. Therefore, the research question is likely even more complex than we’ve alluded to and any future prospective studies should consider including midline and PICC catheters in what is likely to be a complex RCT design. Fourth, and perhaps most challenging may be overcoming the engrained belief among clinicians [42] that vasopressor medications ought to be administered through a CVC and the effects of this belief on clinicians’ willingness to enroll patients into an RCT that potentially randomizes patients to vasopressor administration other than through a CVC.

Though our study highlights the need for additional high-quality research, taken together with existing guidelines, our results help inform current clinical practice with regard to PIV vasopressor administration. The most recent Surviving Sepsis Campaign Guidelines for management of adults with sepsis do not make explicit recommendations regarding CVC versus PIV vasopressor administration, though it is highlighted that patients no longer require assessment of specialized data obtained from the CVC such as central venous oxygen saturation as part of their initial resuscitation [43]. Pediatric sepsis guidelines state that if a CVC is not reasonably accessible, all vasoactive medications (including epinephrine and norepinephrine) can be given through a PIV or IO to avoid delays and to transfer the infusion to a CVC as soon as possible [44]. Our sensitivity analysis excluding the large study in operative patients [11] suggests that adverse events may occur more commonly in patients in ICUs, EDs and/or step-down units where vasopressor infusions are likely administered for longer periods of time to patients with higher illness severity, though the overall rate was still low. The wide variety of studies represented in this review, the majority of which report a low incidence of adverse events, suggest that, for at least short periods of time, PIV vasopressor administration is safe provided precautions exist to reduce the likelihood of adverse events. Such safeguards include institutional policies that place limitations on PIV size and location, infusion dose and duration, require frequent PIV checks to ensure patency and always maintaining a backup PIV in case of sudden loss of intravenous access [2, 3, 10, 40].

The findings of this study should be interpreted in the context of its strengths and limitations. It was conducted by rigorously following methodological guidelines for meta-analyses, and to our knowledge, is the first meta-analysis to examine the safety of PIV vasopressor administration in children, and the largest in adults. However, most included studies were single-centre, retrospective cohort studies, inherently at risk of reporting bias, and were generally at high/some risk of bias. Subgroup and meta-regression analyses suggest a reduction in heterogeneity among those studies at lower risk of bias, however the adverse event estimate remained similar. In addition, there was significant interstudy heterogeneity. Subgroup and stratified analyses examining factors known to contribute to clinical heterogeneity such as location of clinical care, PIV gauge, and vasopressor infusion duration were identified as factors contributing to the observed statistical heterogeneity. Owing to non-uniform reporting of data within the included studies we were unable to comment on contributions of vasopressor concentration and/or dose, two factors predicted to be important determinants of the safety of PIV vasopressor administration. Sensitivity analysis excluding a large study of operative patients by Pancaro et al. wherein data was primarily obtained from an electronic database (rather than manual chart review) and adverse event rates were reported to be lower [11], resulted in a marked reduction in heterogeneity among the residual studies in ICU, ED and step down unit patients. Based on its contribution to heterogeneity, combined with the fact that the study by Pancaro et al. was the only study to obtain adverse event data from a pre-existing electronic database wherein report of more minor adverse events such as extravasation, skin blanching, and mottling may be lower, argument could be made to exclude it from the main meta-analysis. However, we elected to keep it in the main meta-analysis and report its exclusion as a separate sensitivity analysis to be consistent with our original statistical analysis plan, as well as to reflect the true state of current literature. In addition, we employed a random effects model which more evenly distributes study weighting and prevents larger studies such as that of Pancaro et al. from having undue influence on pooled effect estimates. Asymmetry in vasopressor type among the included studies is another potential limitation. Norepinephrine was the most common and this likely reflects clinical practice in most jurisdictions, however, other frequently employed drugs such as epinephrine and vasopressin were underrepresented. Attempts to examine our results in subgroups defined by vasopressor type did not yield any significant differences in the incidence of adverse events, though the analysis was likely underpowered.

## Conclusion

The incidence of adverse events associated with PIV vasopressor administration in adults and children is low. When adverse events did occur, they tended to be minor. Additional research is required to examine the effects of PIV location and size, vasopressor type, concentration, dose and duration, and patient characteristics on the safety of PIV vasopressor administration. However, in the meantime, these results suggest that with careful monitoring, administration of vasopressors through PIVs is safe.

## Supplementary Information

**Additional file 1. Table S1**: MEDLINE search strategy. **Table S2**: Conference abstracts searched. **Table S3**: Demographic and clinical characteristics of patients who received peripheral intravenous vasopressors. **Table S4**: Adverse event characteristics. **Table S5**: Revised Cochrane risk-of-bias tool for randomized trials (RoB 2). **Table S6**: JBI Critical Appraisal Checklist for Studies Reporting Prevalence Data. **Fig. S1**: Funnel plot of studies included in the meta-analysis. **Fig. S2**: Forest plot examining the incidence proportion of adverse events associated with peripheral intravenous vasopressor administration of adult studies stratified by location of care. **Fig. S3**: Forest plot examining the incidence proportion of adverse events associated with peripheral intravenous vasopressor administration of Adult studies stratified by study design. **Fig. S4**: Forest plot examining the incidence proportion of adverse events associated with peripheral intravenous vasopressor administration of adult studies stratified by the mean/median duration of infusion. **Fig. S5**: Forest plot examining the incidence proportion of adverse events associated with peripheral intravenous vasopressor administration of adult studies stratified by Intravenous gauge. **Fig. S6**: Forest plot examining the incidence proportion of adverse events associated with peripheral intravenous vasopressor administration of Adult studies stratified by anatomical location. **Fig. S7**: Forest plot examining the incidence proportion of adverse events associated with peripheral intravenous vasopressor administration of adult studies stratified by vasopressor type. **Fig. S8**: Forest plot examining the incidence proportion of adverse events associated with peripheral intravenous vasopressor administration of adult studies stratified by patient sex. **Fig. S9**: Sensitivity analysis of forest plot examining the incidence proportion of adverse events associated with peripheral intravenous vasopressor administration in adults.

## Data Availability

Original data is available from the corresponding author on reasonable request.

## Abbreviations

ICU: Intensive care units
CVC: Central venous catheter
PIV: Peripheral intravenous
PRISMA: Preferred Reporting Items for Systematic Reviews and Meta-analyses
RoB 2: Revised Cochrane risk-of-bias tool for randomized trials
JBI: Joanna Briggs Institute
IO: Interosseous
RCT: Included randomized control trial
ED: Emergency departments
OR: Operating room
USA: United States of America
