# Supplementary material for: Adverse events associated with administration of vasopressor medications through a peripheral intravenous catheter: a systematic review and meta-analysis
Source: Crit Care. 2021 Apr 16;25:146. doi: 10.1186/s13054-021-03553-1 (PMC8050944; doi:10.1186/s13054-021-03553-1)
Supplement: Supplementary file 1 — Additional file 1. Table S1: MEDLINE search strategy. Table S2: Conference abstracts searched. Table S3: Demographic and clinical characteristics of patients who received peripheral intravenous vasopressors. Table S4: Adverse event characteristics. Table S5: Revised Cochrane risk-of-bias tool for randomized trials (RoB 2). Table S6: JBI Critical Appraisal Checklist for Studies Reporting Prevalence Data. Fig. S1: Funnel plot of studies included in the meta-analysis. Fig. S2: Forest plot examining the incidence proportion of adverse events associated with peripheral intravenous vasopressor administration of adult studies stratified by location of care. Fig. S3: Forest plot examining the incidence proportion of adverse events associated with peripheral intravenous vasopressor administration of Adult studies stratified by study design. Fig. S4: Forest plot examining the incidence proportion of adverse events associated with peripheral intravenous vasopressor administration of adult studies stratified by the mean/median duration of infusion. Fig. S5: Forest plot examining the incidence proportion of adverse events associated with peripheral intravenous vasopressor administration of adult studies stratified by Intravenous gauge. Fig. S6: Forest plot examining the incidence proportion of adverse events associated with peripheral intravenous vasopressor administration of Adult studies stratified by anatomical location. Fig. S7: Forest plot examining the incidence proportion of adverse events associated with peripheral intravenous vasopressor administration of adult studies stratified by vasopressor type. Fig. S8: Forest plot examining the incidence proportion of adverse events associated with peripheral intravenous vasopressor administration of adult studies stratified by patient sex. Fig. S9: Sensitivity analysis of forest plot examining the incidence proportion of adverse events associated with peripheral intravenous vasopressor administration in adults. [file 13054_2021_3553_MOESM1_ESM.docx]

**Supplementary Table 1.**  MEDLINE search strategy

| Class of Medication: Vasopressors | Route of administration: Intravenous | Type of study: cohort, quasi-experimental, or randomized controlled trials | All: removing Animal only studies and case reports. |
| --- | --- | --- | --- |
| 1 exp Vasoconstrictor Agents/  2 Calcium Channel Agonists/  3 exp arginine vasopressin/  4 arginine vasopressin.mp.  5 exp Dopamine Agents/  6 exp Dopamine Agonists/  7 Dopamine.mp.  8 exp Ephedrine/  9 Ephedrine.mp.  10 exp Epinephrine/  11 Epinephrine.mp.  12 exp Felypressin/  13 Felypressin.mp.  14 exp Lypressin/  15 Lypressin.mp.  16 exp Midodrine/  17 Midodrine.mp.  18 exp Norepinephrine/  19 Norepinephrine.mp.  20 exp Ornipressin/  21 (Pitressin or "beta hypophamine" or orpressin).mp. (560)  22 exp Phenylephrine/  23 Phenylephrine.mp.  24 exp Racepinephrine/  25 Racepinephrine.mp.  26 exp Synephrine/  27 Synephrine.mp.  28 exp Terlipressin/  29 Terlipressin.mp.  30 exp Vasopressins/  31 vasopressin*.mp.  32 (vasopressor* or vasoactive agonist*).mp.  33 vasoactive medication*.mp.  34 or/1-33 | 35 exp Central Venous Catheters/  36 exp Catheterization, Central Venous/  37 Catheterization/  38 limit 37 to yr="1966-1987"  39 (central* adj2 (line or lines or IV or catheter* or venous or intravenous or infusion* or administ*)).mp.  40 Administration, Intravenous/  41 exp Infusions, Intravenous/  42 exp Catheterization, Peripheral/  43 (peripheral* adj2 (intravenous or IV or venous or line or lines or catheter* or infusion* or administ*)).mp.  44 35 or 36 or 38 or 39 or 40 or 41 or 42 or 43 | 45 "randomized controlled trial".pt.  46 (random$ or placebo$ or single blind$ or double blind$ or triple blind$).ti,ab.  47 (retraction of publication or retracted publication).pt.  48 or/45-47  49 (animals not humans).sh.  50 ((comment or editorial or meta-analysis or practice-guideline or review or letter) not "randomized controlled  trial").pt.  51 (random sampl$ or random digit$ or random effect$ or random survey or random regression).ti,ab. not "randomized  controlled trial".pt.  52 48 not (49 or 50 or 51)  53 exp cohort studies/  54 cohort$.tw.  55 controlled clinical trial.pt.  56 epidemiologic methods/  57 limit 56 to yr=1971-1988  58 53 or 54 or 55 or 57  59 52 or 58  60 (medical record* adj2 (clinical or electronic)).mp.  61 administrative data*.mp.  62 chart review*.mp.  63 (quasi-experimental or quasiexperimental or quasi experimental).mp.  64 exp Observation/  65 monitor*.mp.  66 epidemiologic studies/  67 Controlled Before-After Studies/  68 Historically Controlled Study/  69 incidence/  70 incidence.mp.  71 cohort.ti,ab.  72 longitudinal.ti,ab.  73 prospective.ti,ab.  74 retrospective.ti,ab.  75 59 or 60 or 61 or 62 or 63 or 64 or 65 or 66 or 67 or 68 or 69 or 70 or 71 or 72 or 73 or 74 | 76 34 and 44 and 75  77 limit 76 to animals  78 limit 77 to (animals and humans)  79 77 not 78  80 76 not 79  81 limit 80 to case reports  82 80 not 81 |

**Supplementary Table 2.** Conference abstracts searched.

| **Conference** | **2017** | **2018** | **2019** |
| --- | --- | --- | --- |
| American Society of Anesthesiologists | Searched | Searched | Searched |
| Canadian Anesthesiologists' Society Annual Meeting | Unable to locate | Searched | Searched |
| Canadian Critical Care Forum | Searched | Searched | Searched |
| European Society of Intensive Care Medicine | Searched | Searched | Searched |
| European Society of Anaesthesia Annual Meeting (Euroanaesthesia) | Searched | Searched | Searched |
| European Congress on Emergency Medicine | Unable to locate | Unable to locate | Searched |

**Supplementary Table 3.** Demographic and clinical characteristics of patients who received peripheral intravenous vasopressors.

| Author (year) | Patients  Received PIV vasopressors, n | Mean (SD) Age (years) | Male Sex, n(%) | Illness severity measure | Reason For PIV vasopressor, n(%) | Vasopressor Medication,  n(%) | Vasopressor Dose | Hours of Vasopressor Infusion | Intravenous Gauge, n (%) | Intravenous Location, n(%) | Transition to CVC, n(%) | Adverse Events, n | Intervention for Adverse Event |  |
| --- | --- | --- | --- | --- | --- | --- | --- | --- | --- | --- | --- | --- | --- | --- |
| Studies in Adults | | | | | | | | | | | | | |  |
| Andrews (2017) (1) | 17*^a^* | NR | NR | NR | Sepsis | Dopamine 17(100%)*^a^* | >10 mcg/kg/min | NR | NR | NR | No | None*^a^* | NA |  |
| Johnson, W. (1977) (2) | 11 | NR | NR | 30 day Mortality 45% | Hemorrhagic shock due to esophagogastic varices | Vasopressin 11(100%) | 0.1-0.4 units/min | >48 hours*^b^* | NR | NR | No | Phlebitis (1) | NR |  |
| Medlej  (2018) (3) | 55 | 70(NR) | 34 (62%) | In hospital mortality 32% | Shock – mixed etiologies | Norepinephrine 50(91%)*^c^*;  Dopamine 5(9%)*^c^* | Maximum infusion rate: Norepinephrine 30 mcg/min; Dopamine 15 mcg/kg/min | Median (IQR)  All 14 (7–40);  Norepinephrine 13 (6.5-31.5);  Dopamine 53 (15.5-113) | 16G 6(11%);  18G 20(36%);  20G 28(51%);  22G 1(2%) | External jugular 2(4%);  Upper arm 1(2%);  ACF 22(40%);  Forearm 10(18%);  Hand 20(36%) | 13 (24%) | Drug extravasation causing:  skin parlour (1);  non blanching skin erythema(1);  thrombophlebitis(1) | None |  |
| Lewis  (2019) (4) | 202 | Median (IQR) 75 (64-83) | 107(53%) | APACHE-II  Median (IQR)  21(17-25) | Primarily for Shock – mixed etiologies | Norepinephrine 146 (72%)*^d^*;  phenylephrine 73(36%)*^d^*;  vasopressin  4(2%)*^d^*;  epinephrine 2(1%)*^d^*;  dopamine 2(1%)*^d^* | Median (IQR):  norepinephrine 0.08 (0.05-0.17) mcg/kg/min;  phenylephrine 50 (25-100) mcg/min  vasopressin  0.04(NR) units/min;  epinephrine 0.05(NR) mcg/kg/min:  dopamine 8(NR) mcg/kg/min | Median(IQR) All: 11.5(NR);  norepinephrine 7.5 (3-23) ;  phenylephrine 15 (6-38);  vasopressin  12.5 (4-23) ;  epinephrine 4.5(NR)  dopamine 23.5(NR) | 18G 46(23%)*^d^*; 20G 149(74%)*^d^*;  22G 103(51%)*^d^*; other 6(3%)*^d^* | Forearm 145(72%)*^d^*;  ACF 109(54%)*^d^*;  hand 81(40%)*^d^*;  other 5(2%)*^d^* | 93 (46%) | Extravasation causing: blanching(5),  Blanching and edema(3), | Removal of PIV where extravasation occurred |  |
| Pancaro  (2019) (5) | 14 385 | NR | NR | NR | Anesthetic-related hypotension | Norepinephrine 14 385 (100%) | Typically from 0.01-0.1mcg/kg/min | NR | NR | NR | NR | Extravasation (5) | Stop infusion and monitor |  |
| Datar  (2018) (6) | 277 | 65(15) | 129(47%) | NR | Hemodynamic augmentation 110 (40%);  hypotension, 167 (60%) | Phenylephrine 277(100%) | Mean (SD) maximum rate, 1.04 (0.74) mcg/kg/min | Mean (SD) 19(18) | 16G 13(5%);  18G 98(35%);  20G 113(41%);  22G 5(2%);  Unknown 48(17%) | Upper extremity 139(50%);  wrist/hand 87(32%);  other 3(1%);  unknown 48(17%) | 57(21%)*^e^* | Infiltration (9) | None |  |
| Hallengren (2017) (7) | 79 | Median (IQR) 81(73, 88)*^f^* | 50(63%)*^f^* | Median(Q1 Q3): APACHE II 26(23,32)*^f^* | Sepsis | Norepinephrine 79(100%) | Maximum rate 0.2 mcg/kg/min*^b^* | Median (Q1, Q3) 13 (8,30)*^f^* | NR | NR | 2(2.5%) patients had difficulty with PIV access and had CVCs inserted | None | NA |  |
| Delgado  (2016) (8) | 20 | Mean (range) 62 (14-90) | 11(55%) | NR | Hemodynamic support or augmentation | Phenylephrine 20(100%) | Mean dose(range) 0.53 (0.19-1.84) mcg/kg/min | Mean (range) 14.3 (1-54.3) | 18G or larger 19(95%);  20G 1(5%) | Upper extremity (proximal to wrist); 20(100%)*^b^* | NR | Pain, erythema, swelling, and need to replace IV (1) | Replacement of IV |  |
| Cardenas-Garcia  (2015) (9) | 734 | 72(15) | 398 (54%) | Mean (SD)  SAPS-II  75(15) | Clinical management of medical ICU patients. | Norepinephrine 506(65%)*^d^*;  dopamine 101(13%)*^d^*;  phenylephrine 176(22%)*^d^* | Mean (SD) in mcg/kg/min:  norepinephrine  0.70 (0.23)*^d^*;  dopamine 12.7 (5.23)*^d^*;  phenylephrine 3.25 (1.69)*^d^* | Mean (SD) 49 (22) | 18G 192(25%)*^d^*;  20G 590(75%)*^d^*;  22G 1(0%)*^d^* | Not formally recorded, but most in upper arm | 95 (13%) | Extravasation (19) | Phentolamine injection and application of nitroglycerin paste at extravasation site |  |
| Putland  (2006) (10) | 220 | Mean (range) 36 (18-55) | 88(40%) | Australian triage scale:  category 1: 46(21%)  category 2: 129(59%)  category 3: 45(20%) | Asthma | Epinephrine 220 (100%) | Mean (range) 1.5 (0.5 to 13.3) mcg/min | Median (range) 19.5 (0.17- 273.6) | NR | NR | NR | Local tissue pallor or mottling (11 events in 7 patients) | NR |  |
| Rojewski-Rojas  (2019)^g^ (11) | 55 | 73.6 (13.9) | 25 (46%) | Mortality 37% | Shock – mixed etiologies | Norepinephrine 53(96%);  epinephrine 1(2%); dopamine 1(2%) | NR | Median 24(NR) | NR | NR | NR | Complications(2) | NR |  |
| Delaney  (2019) (12) | 389 | Median (IQR) 65.4 (52.4, 75.3) | 233(60%) | Median (IQR)  APACHE-II  17(13,23) | Septic shock | Norepinephrine 293(75%);  epinephrine 38(10%);  metaraminol 58(15%) | NR | Median(IQR) 1.3 (0.66, 2.5) | NR | NR | 360 (92.5%) | NR | NA |  |
| White  (2001) (13) | 13*^h^* | NR | NR | NR | Clinical management of hospital patients. | Dopamine (NR) | NR | NR | NR | NR | NR | Phlebitis (1) | NR |  |
| Dugger  (1997) (14) | 25*^d^* | NR | NR | NR | Renal perfusion augmentation (95%) | Dopamine 25(100%)*^d^* | 1 to 5 mcg/kg/min (in 95% of cases) | NR | 18G 1(4%)  20G 7(28%)  22G 7(28%)  24G 1(4%)  Missing data 9(36%) | Usually in forearms | NR | Extravasation (17) | Phentolamine injected into the infiltrated catheter and the surrounding subcutaneous tissue. |  |
| Zucker  (1960) (15) | 68 | NR | NR | Mortality 48(70.6%) | Shock – mixed etiologies | Norepinephrine 68 (100%) | Norepinephrine range 0.08 - 0.96 mcg/Kg/minute | Range 1-264 | NR | Forearm (NR);  antecubital fossa (NR);  hand (NR);  leg (NR) | NR | Extravasations (34 events in 22 patients) causing slough (1) | Phentolamine added to the norepinephrine infusions. |  |
| Moyer  (1955) (16) | 20 | 63(19.3)*^f^* | 14(70%) | Mortality 14 (70%) | Shock – mixed etiologies | Metaraminol 20(100%); norepinephrine and metaraminol 4(20%) | metaraminol range 2- 6 c.c./min | Median (IQR) 26(15, 51.5)*^f^* | NR | NR | Yes (%NR) | Extravasations (‘several’); pain at insertion site(1) | NR |  |
| Studies in Children | | | | | | | | | | | | | |  |
| Kumar (2015) (17) | 190 | 120 (58.8%)*^i^* infants, 56 (27.5%)*^i^* 1- 5 years, 21 (10.3%)*^i^* 6 - 10 years 7 (3.4%)*^i^* > 10 years | 109(53%)*^i^* | Mortality 100(49%)*^i^* | Shock – mixed etiologies | Dopamine 172(84%)*^j^*;  epinephrine 49(24%)*^j^*;  norepinephrine 62(30%)*^j^* | Range 10–20 mcg/kg/min*^b^* for dopamine and 0.1-1 mcg/kg/min*^b^* for epinephrine and norepinephrine. | Mean(SD)) norepinephrine 11.9(8.9); dopamine 31.4 (41); epinephrine 16.6 (16.9) | NR | Hand(NR)  Forearm(NR)  ACF(NR)  Foot(NR) | No | Extravasation (3) | NR |  |
| Patregnani (2017) (18) | 102 | Median (IQR) 9.3 (3.8-15.3) | 52(51%) | PIM III, % (IQR)  1.4 (0.8–4.8) | Shock – mixed etiologies and hemodynamic augmentation | Dopamine 87(85%)*^j^*;  epinephrine 26(25%)*^j^*;  norepinephrine 11(11%)*^j^*;  vasopressin 6(6%)*^j^* | Median (IQR) Peak Vasoactive Infusion Score 10 (6–14)*^k^* | Median (IQR)  4.3(2.4-9) | 16G 1(1%);  18G 9(9%);  20G 20(20%);  22G 32(31%);  24G 40(39%); | NR | 64(63%) | PIV infiltration and extravasation  with tissue edema (2) | NR |  |
| Turner  (2010) (19) | 73 | Median (range) 1 day (birth to 19 years) | 40 (55%) | NR | Clinical management | Dopamine 70 (96%)*^j^*;  epinephrine 6 (8%)*^j^*; phenylephrine 1(1%)*^j^*. | Median (range) maximum dose Dopamine 10 (1 to 22.5) mcg/kg/min | Median (range) 5.3 (0.17-116.8) | Median (range) 24 (18-24) | NR | Yes, (%NR). | Infiltration (involving minimal blanching and/or erythema)(11)*^l^* | None |  |
| Lampin  (2012) (20) | 23*^m^* | NR | NR | NR | Septic shock | Dopamine  62(42%)*^n^*;  Norepinephrine 23(16%) | NR | Median (IQR)  Norepinephrine  3 (2–4)*^n^* ;  Dopamine NR | NR | NR | Yes, (%NR) | Extravasation causing Ischemia and skin necrosis from PIV dopamine (1) | NR |  |
| Ventura  (2015) (21) | 120*^n^* | Mean(SD)3.98(4.4)*^n,o^* | 70 (58%)*^n^* | Mean(SD) Pediatric Risk of Mortality 15.1 (10.1)*^n,o^* | Septic shock | Dopamine 63(52.5%)*^n^*;  epinephrine 57 (47.5%)*^n^* | NR | Mean(SD) Dopamine 20.4 (21.4)*^n^*;  epinephrine 36.5 (46.3)*^n^* | NR | NR | Yes, n not reported. | None | NA |  |
| Stanley  (1992) (22) | NR | NR | NR | NR | Clinical management of NICU patients. | Dopamine (NR)*^p^* | NR | NR^p^ | 24G (100%) | NR^p^ | NR | Infiltrates (NR) | Catheter was removed if complication occurred |  |
| Johnson, R. (1988) (23) | NR | NR | NR | NR | Clinical management of NICU patients. | Dopamine (NR)*^p^* | NR | NR^p^ | 24G(all but one) | NR | No | Infiltration causing sloughing (1) | Topical antibiotics |  |

**Abbreviations:** PIV=peripheral intravenous; CVC=central venous catheter; AE=averse events; NA=not applicable; PIM=Pediatric Index of Mortality; NR=none reported; SAPS II, simplified acute physiology score II; ACF= antecubital fossa, SAPS II= simplified acute physiology score II, APCHE II= Acute Physiology and Chronic Health Evaluation

**Superscripts:**

a= Adverse events were only monitored for within the first 6 hours of admission to ED

b= Implied

c= Three of the 55 patient’s received two vasopressors through their PIV, none of the patients that received multiple infusions experienced adverse events

d= Number of infusions or PIVs not number of patients

e= Of those transitioned to CVC, 29(11%) were due to physician preference and 28(10%) were due to addition of another vasopressor not approved for PIV at this institution.

f= statistics could be calculated from data within study

g= Study abstract with poster only. Data preferentially taken from poster.

h= Study contains multiple drugs and it is not explicit how many patient received vasopressors, it was implied to be 13 or less.

i= Summary statistics contain 14 patients who exclusively received dobutamine (not classified as a vasopressor).

j= Some patients received multiple vasopressors, so percentage is greater than 100%.

k= Vasopressor Infusion Score (VIS) was calculated by: dopamine dose (μg/kg/min) + 100 × epinephrine dose (μg/kg/min) + 10,000 × vasopressin dose (U/kg/min) + 100 × norepinephrine dose (μg/kg/min)

l= Iinfiltrations reported in the study did not happen during transport, but after arrival in PICU

m= Study does not report how many patients received dopamine through a PIV (as opposed to IO) so numbers reported are only for epinephrine.

n= Combines patients who received vasopressor through a PIV or IO route.

o= Calculated by combining the two study arms with the Stata function ‘Combine’(24)

p= Study examined multiple drugs and results are not stratified by vasopressors

**Supplementary Table 4.** Adverse event characteristics

| Author (year) | Total Patients Experienced Adverse Events N(%) | Patient Age (years) | Male Sex, n(%) | PIV Gauge, n (%) | PIV Location, n (%) | Vasopressor Dose | Hours of Vasopressor Infusion | Adverse event incidence proportion per vasopressor medication (number of adverse events/total infusions per drug in overall study population) | | | | | |
| --- | --- | --- | --- | --- | --- | --- | --- | --- | --- | --- | --- | --- | --- |
|  | | | | | | | | Norepinephrine | Dopamine | Epinephrine | Phenylephrine | Vasopressin | Metaraminol |
| Studies in Adults | | | | | | | | | | | | | |
| Andrews (2017) (1) | 0 | -- | -- | -- | -- | -- | -- | -- | 0% (0/17)*^a^* | -- | -- | -- | -- |
| Johnson, W.  (1977) (2) | 1 | NR | NR | NR | Forearm 1(100%) | NR | NR | -- | -- | -- | -- | Phlebitis 9% (1/11) | -- |
| Medlej (2018) (3) | 3 | 42, 57 and 58 | 1 (33%) | 20G 3 (100%) | Hand. 2 (67%);  ACF, 1 (33%) | Maximal dose:  7 mcg/min, 2 (67%);  19 mcg/min, 1 (33%) | Norepinephrine:  11, 28 and 40 | Drug extravasation 6% (3/50)  causing: skin parlour (1/50); non blanching skin erythema(1/50);  thrombophlebitis(1/50) | 0% (0/5) | -- | -- | -- | -- |
| Lewis (2019) (4) | 8 | Median(IQR) 75 (69.5-89)*^b^* | 3 (37.5%)*^b^* | <20G, 2(25%);  >20G, 6 (75%) | Hand, 2(25%);  ACF, 2(25%);  other, 4(50%) | Median dose  at time of extravasation 0.11 mg/kg/min*^c^* | Median (IQR)  21 (12-30) | Extravasation: 2.7%  (4/146) | 0% (0/2) | 0%, (0/2) | Extravasation: 5.5% (4/73) | 0% (0/4) |  |
| Pancaro (2019) (5) | 5 | Mean(SD) 66.2 (18.4) | 2 (40%) | Median (IQR) 18 (18,18) | Hand, 1 (20%);  ACF, 3 (60%);  Lower extremities, 1 (20%) | Dose range 0.02-0.05mcg/kg/min | Median (IQR)  0.33 (0.33–0.42) | Extravasation: 0.03%  (5/14 385) | -- | -- | -- | -- | -- |
| Datar (2018) (6) | 9 | NR | 5 (56%)*^b^* | NR | -- | NR | NR | -- | -- | -- | Infiltration: 3.2%  (9/277) | -- | -- |
| Hallengren (2017) (7) | 0 | -- | -- | -- | -- | -- | -- | 0% (0/79) | -- | -- | -- | -- | -- |
| Delgado (2016) (8) | 1 | 49 | 0 (0%) | 18G, 1 (100%) | ACF, 1 (100%) | Peak dose 0.35mcg/kg/min  Mean dose 0.33 mcg/kg/min | 17.27 | -- | -- | -- | Pain, erythema, swelling, and need to replace IV: 5% (1/20) | -- | -- |
| Cardenas-Garcia (2015) (9) | 19 | NR | NR | <20G, 1(5%);  >20G 18 (95%) | NR | NR | NR | Extravasation: 3.2% (16/506) | Extravasations: 3.0% (3/101) | -- | 0% (0/176) | -- | -- |
| Putland (2006) (10) | 7*^d^* | NR | NR | NR | NR | NR | NR | -- | -- | Local tissue pallor or mottling: 5.0% (11/220) | -- | -- | -- |
| Rojewski-Rojas (2019) (11) | 2 | NR | NR | NR | NR | NR | NR | NR/53 *^e^* | NR/1 *^e^* | NR/1 *^e^* | -- | -- | -- |
| Delaney (2019) (12) | --*^f^* | NR | NR | NR | NR | NR | NR | NR/293 *^f^* | -- | NR/38 *^f^* | -- | -- | NR/58 *^f^* |
| White (2001) (13) | 1 | NR | NR | NR | NR | NR | NR | -- | Phlebitis 1/--^g^ | -- | -- | -- | -- |
| Dugger (1997) (14) | 17*^h^* | NR | NR | NR | NR | NR | Mean 9.7 | -- | Extravasation: 68% (17/25)*^h^* | -- | -- | -- | -- |
| Zucker (1960) (15) | 22*^i^* | NR | NR | NR | NR | NR | NR | Extravasation: 50% (34/68)  Skin slough: 1.5% (1/68) | -- | -- | -- | -- | -- |
| Moyer (1955) (16) | --*^j^* | NR | NR | NR | NR | NR | NR | NR/4*^j^* | -- | -- | -- | -- | extravasation NR/20*^j^*  Pain at insertion site 5% 1/20 |
| Studies in Children | | | | | | | | | | | | | |
| Kumar (2015) (17) | 3 | NR | NR | NR | NR | NR | NR | 0% (0/62)*^k^* | Extravasation 0.6% (1/172)*^k^* | Extravasation 4% (2/49)*^k^* | -- | -- | -- |
| Patregnani (2017) (18) | 2 | NR | NR | NR | Distal extremity, 2 (100%) | NR | Less than 2 hours | (NR*^e^*/11*^k^*) | (NR*^e^*/87*^k^*) | (NR*^e^*/26*^k^*) | -- | (NR*^e^*/6*^k^*) | -- |
| Turner (2010) (19) | 11 | Median 1 day | 9 (82%) | Median 24G | NR | median (range) Dopamine: 15 (8-22.5 ) mcg/kg/min | Mean(SD) 17.8(3.9) | -- | (NR*^e^*/70*^k^*) | (NR*^e^*/6*^k^*) | (NR*^e^*/1*^k^*) | -- | -- |
| Lampin (2012) (20) | 1*^l^* | NR | NR | NR | NR | NR | NR | 0% (0/23) | Ischemia and skin necrosis (1/NR*^l^*) | -- | -- | -- | -- |
| Ventura (2015) (21) | 0 | -- | -- | -- | -- | -- | -- | -- | (0/NR*^g^*) | (0/NR*^g^*) | -- | -- | -- |
| Stanley (1992) (22) | --*^m^* | NR | NR | NR | NR | NR | NR | -- | (NR*^e^*/NR*^g^*) | -- | -- | -- | -- |
| Johnson, R. (1988) (23) | 1 | NR | NR | NR | NR | NR | 47 | -- | (1/NR*^g^*) | -- | -- | -- | -- |

**Abbreviations:** ACF=antecubital fossa; NR=not reported; --=not applicable

**Superscripts:**

a = More patients received PIV vasopressors, but adverse events were only monitored in 17 patients and only for the first 6 hours of the infusion.

b = Data made available following contact with study authors

c = Measured in norepinephrine equivalents, calculated by: (norepinephrine [mcg/min]) + (dopamine [mcg/kg/min]/2) + (epinephrine [mcg/min]) + (phenylephrine [mcg/min]/10) + (vasopressin [units/h] x 8.33).(4)

d = 11 cases of local tissue ischemia occurred in 7 patients (5 in same patient)

e = Study reports multiple drugs and does not stratify adverse events by vasopressors.

f = Adverse events associated with PIV vasopressor administration not prospectively recorded in original RCT(25)

g = Study is unclear how many patients received PIV vasopressors.

h = Number of infusions not number of patients

i = 34 extravasations happened in 22 patients

j = Unable to calculate number of adverse events based on data reported in manuscript

k = multiple patients received more than one vasopressor, so total is greater than n of patients.

l = Adverse event was in a patient that received dopamine and study did not report how many patients received PIV dopamine so number was not included in Meta-analysis

m= Study reports that IVs with dopamine were 0.59 times the risk for infiltration relative to other infusates, but does not give number of infiltrations per drug.

**Supplementary Table 5.** Revised Cochrane risk-of-bias tool for randomized trials (RoB 2)

| STUDY | Domain 1 | Domain 2 | Domain 3 | Domain 4 | Domain 5 | **Total** |
| --- | --- | --- | --- | --- | --- | --- |
| **Studies in Adults** | | | | | | |
| **Andrews (2017)** (1) | Low | Low | Low | High | Low | **High** |
| **Johnson, W. (1977)** (2) | Low | Low | Low | Low | Some | **Some concerns** |
| **Studies in Children** | | | | | | |
| **Ventura (2015)** (21) | Low | Low | Low | Low | Some | **Some concerns** |
| **Stanley (1992)** (22) | High | Low | Low | Low | Some | **High** |

Low= Low risk of bias in that domain, some= some concerns in that domain, High= High risk of bias in that domain

Domains of Revised Cochrane risk-of-bias tool for randomized trials:

Domain 1: Risk of bias arising from the randomization process

Domain 2: Risk of bias due to deviations from the intended interventions

Domain 3: Risk of bias due to missing outcome data

Domain 4: Risk of bias in measurement of the outcome

Domain 5: Risk of bias in selection of the reported result (26)(p.4)

*Risk of bias was assessed biased on the outcome of interest of this review (adverse events with PIV vasopressors) and not necessarily the primary outcome of the study.

**Supplementary Table 6.** JBI Critical Appraisal Checklist for Studies Reporting Prevalence Data

| Study | Q1 | Q2 | Q3 | Q4 | Q5 | Q6 | Q7 | Q8 | Q9 | Total |
| --- | --- | --- | --- | --- | --- | --- | --- | --- | --- | --- |
| Studies in Adults | | | | | | | | | | |
| **Medlej (2018)** (3) | Y | Y | N/A | Y | U | U | Y | N | U | 4 |
| **Lewis (2019)** (4) | Y | Y | N/A | Y | Y | Y | U | N | Y | 6 |
| **Pancaro (2019)** (5) | Y | Y | N/A | N | Y | U | U | Y | Y | 5 |
| **Datar (2018)** (6) | Y | Y | N/A | Y | Y | U | U | N | Y | 5 |
| **Hallengren (2017)** (7) | Y | Y | N/A | Y | Y | U | U | N | Y | 5 |
| **Delgado (2016)^f^** (8) | Y | Y | N/A | Y | Y | U | Y | N | Y | 6 |
| **Cardenas-Garcia (2015)** (9) | Y | Y | N/A | Y | Y | U | Y | N | Y | 6 |
| **Putland (2006)** (10) | Y | Y | N/A | Y | U | U | U | Y | Y | 5 |
| **Rojewski-Rojas (2019)** (11) | Y | Y | N/A | Y | Y | U | U | N | Y | 5 |
| **Delaney (2019)** (12) | Y | Y | N/A | Y | Y | N/A | N/A | Y | Y | 6 |
| **White (2001)** (13) | Y | Y | N/A | Y | Y | Y | Y | N | Y | 7 |
| **Dugger (1997)** (14) | Y | Y | N/A | N | U | U | U | N | Y | 3 |
| **Zucker (1960)** (15) | Y | U | N/A | N | Y | U | U | N | U | 2 |
| **Moyer (1955)** (16) | Y | U | N/A | N | Y | U | U | N | U | 2 |
| Studies in Children | | | | | | | | | | |
| **Kumar (2015)** (17) | Y | Y | N/A | Y | Y | U | U | N | Y | 5 |
| **Patregnani (2017)** (18) | Y | Y | N/A | Y | Y | U | U | N | Y | 5 |
| **Turner (2010)** (19) | Y | Y | N/A | Y | Y | U | Y | N | Y | 6 |
| **Lampin (2012)** (20) | Y | Y | N/A | Y | Y | U | U | N | Y | 5 |
| **Johnson, R. (1988)** (23) | Y | Y | N/A | N | U | U | U | N | Y | 3 |

Questions from JBI Critical Appraisal Checklist for Studies Reporting Prevalence Data

1. Was the sample frame appropriate to address the target population?

2. Were study participants sampled in an appropriate way?

3. Was the sample size adequate?

4. Were the study subjects and the setting described in detail?

5. Was the data analysis conducted with sufficient coverage of the identified sample?

6. Were valid methods used for the identification of the condition?

7. Was the condition measured in a standard, reliable way for all participants?

8. Was there appropriate statistical analysis?

9. Was the response rate adequate, and if not, was the low response rate managed appropriately? (27)(p.150)

*Risk of bias was assessed biased on the outcome of interest of this review (adverse events with PIV vasopressors) and not necessarily the primary outcome of the study.

**Supplementary Figure 1:** Funnel plot of studies included in the meta-analysis

**Supplementary Figure 2:** Forest plot examining the incidence proportion of adverse events associated with peripheral intravenous vasopressor administration of adult studies stratified by location of care

**Supplementary Figure 3:** Forest plot examining the incidence proportion of adverse events associated with peripheral intravenous vasopressor administration of adult studies stratified by study design

**Supplementary Figure 4:** Forest plot examining the incidence proportion of adverse events associated with peripheral intravenous vasopressor administration of adult studies stratified by the mean/median duration of infusion

*For Greater or equal to 24 hours the subtotal *I^2^* = 5.31%, p = 0.35

**Supplementary Figure 5**: Forest plot examining the incidence proportion of adverse events associated with peripheral intravenous vasopressor administration of adult studies stratified by Intravenous gauge

*For 22G the subtotal *I^2^* = 69.17 %, p = 0.04

**Supplementary Figure 6**: Forest plot examining the incidence proportion of adverse events associated with peripheral intravenous vasopressor administration of Adult studies stratified by anatomical location

*For Proximal to the wrist the subtotal *I^2^* = 39.04%, p = 0.19

**Supplementary Figure 7**: Forest plot examining the incidence proportion of adverse events associated with peripheral intravenous vasopressor administration of adult studies stratified by vasopressor type

**Supplementary Figure 8:** Forest plot examining the incidence proportion of adverse events associated with peripheral intravenous vasopressor administration of adult studies stratified by patient sex

**Supplementary Figure 9:** Sensitivity analysis of forest plot examining the incidence proportion of adverse events associated with peripheral intravenous vasopressor administration in adults

References

1. Andrews B, Semler MW, Muchemwa L, Kelly P, Lakhi S, Heimburger DC, et al. Effect of an Early Resuscitation Protocol on In-hospital Mortality Among Adults With Sepsis and Hypotension: A Randomized Clinical Trial. JAMA. 2017;318(13):1233-40.

2. Johnson WC, Widrich WC, Ansell JE, Robbins AH, Nabseth DC. Control of bleeding varices by vasopressin: a prospective randomized study. Ann Surg. 1977;186(3):369-76.

3. Medlej K, Kazzi AA, El Hajj Chehade A, Saad Eldine M, Chami A, Bachir R, et al. Complications from Administration of Vasopressors Through Peripheral Venous Catheters: An Observational Study. J Emerg Med. 2018;54(1):47-53.

4. Lewis T, Merchan C, Altshuler D, Papadopoulos J. Safety of the Peripheral Administration of Vasopressor Agents. J Intensive Care Med. 2019;34(1):26-33.

5. Pancaro C, Shah N, Pasma W, Saager L, Cassidy R, van Klei W, et al. Risk of Major Complications After Perioperative Norepinephrine Infusion Through Peripheral Intravenous Lines in a Multicenter Study. Anesth Analg. 2020;131(4):1060-5.

6. Datar S, Gutierrez E, Schertz A, Vachharajani V. Safety of Phenylephrine Infusion Through Peripheral Intravenous Catheter in the Neurological Intensive Care Unit. J Intensive Care Med. 2018;33(10):589-92.

7. Hallengren M, Astrand P, Eksborg S, Barle H, Frostell C. Septic shock and the use of norepinephrine in an intermediate care unit: Mortality and adverse events. PLoS ONE. 2017;12(8):e0183073.

8. Delgado T, Wolfe B, Davis G, Ansari S. Safety of peripheral administration of phenylephrine in a neurologic intensive care unit: A pilot study. J Crit Care. 2016;34:107-10.

9. Cardenas-Garcia J, Schaub KF, Belchikov YG, Narasimhan M, Koenig SJ, Mayo PH. Safety of peripheral intravenous administration of vasoactive medication. J Hosp Med. 2015;10(9):581-5.

10. Putland M, Kerr D, Kelly AM. Adverse events associated with the use of intravenous epinephrine in emergency department patients presenting with severe asthma. Ann Emerg Med. 2006;47(6):559-63.

11. Rojewski-Rojas W, Alvarez-Galarraga A, Garcia R, Guerra F, editors. A case series on vasoactive drugs via peripheral venous access for management of shock in the emergency room. EUSEM: The European Emergency Medicine Congress; 2019 2019; Prague2019.

12. Delaney A, Finnis M, Bellomo R, Udy A, Jones D, Keijzers G, et al. Initiation of vasopressor infusions via peripheral versus central access in patients with early septic shock: A retrospective cohort study. Emerg Med Australas. 2020;32(2):210-9.

13. White SA. Peripheral intravenous therapy-related phlebitis rates in an adult population. J Intraven Nurs. 2001;24(1):19-24.

14. Dugger B. Peripheral dopamine infusions: are they worth the risk of infiltration? J Intraven Nurs. 1997;20(2):95-9.

15. Zucker G, Eisinger RP, Floch MH, Singer MM. Treatment of shock and prevention of ischemic necrosis with levarterenol-phentolamine mixtures. Circulation. 1960;22:935-7.

16. Moyer JH, Beazley HL. Effectiveness of aramine in the treatment of shock. American Heart Journal. 1955;50:136-44.

17. Kumar S, Varadarajan P, Sangareddi S. Study of Vasoactive Infusions through Peripheral Line. Pediatric Oncall 2015;12(2):31-3.

18. Patregnani JT, Sochet AA, Klugman D. Short-Term Peripheral Vasoactive Infusions in Pediatrics: Where Is the Harm? Pediatr Crit Care Med. 2017;18(8):e378-e81.

19. Turner DA, Kleinman ME. The Use of Vasoactive Agents Via Peripheral Intravenous Access During Transport of Critically Ill Infants and Children. Pediatric Emergency Care. 2010;26(Number 8):563-6.

20. Lampin ME, Rousseaux J, Botte A, Sadik A, Cremer R, Leclerc F. Noradrenaline use for septic shock in children: doses, routes of administration and complications. Acta Paediatr. 2012;101(9):e426-30.

21. Ventura AM, Shieh HH, Bousso A, Goes PF, de Cassia FOFI, de Souza DC, et al. Double-Blind Prospective Randomized Controlled Trial of Dopamine Versus Epinephrine as First-Line Vasoactive Drugs in Pediatric Septic Shock. Crit Care Med. 2015;43(11):2292-302.

22. Stanley MD, Meister E, Fuschuber K. Infiltration during intravenous therapy in neonates: comparison of Teflon and Vialon catheters. South Med J. 1992;85(9):883-6.

23. Johnson RV, Donn SM. Life span of intravenous cannulas in a neonatal intensive care unit. Am J Dis Child. 1988;142(9):968-71.

24. Jones PM. COMBINE: Stata module to combine n, mean, and SD from two groups according to the Cochrane-recommended formula for meta-analyses: Statistical Software Components S457265, Boston College Department of Economics; 2011 [cited 2020 June 22]. Available from: https://ideas.repec.org/c/boc/bocode/s457265.html.

25. Udy AA, Finnis M, Jones D, Delaney A, Macdonald S, Bellomo R, et al. Incidence, Patient Characteristics, Mode of Drug Delivery, and Outcomes of Septic Shock Patients Treated With Vasopressors in the Arise Trial. Shock. 2019;52(4):400-7.

26. Sterne JAC, Savovic J, Page MJ, Elbers RG, Blencowe NS, Boutron I, et al. RoB 2: a revised tool for assessing risk of bias in randomised trials. BMJ. 2019;366:l4898.

27. Munn Z, Moola S, Lisy K, Riitano D, Tufanaru C. Methodological guidance for systematic reviews of observational epidemiological studies reporting prevalence and cumulative incidence data. Int J Evid Based Healthc. 2015;13(3):147-53.
